# The contribution of medically assisted reproduction to total, age-, and parity-specific fertility in Italy

**DOI:** 10.1093/humrep/deaf137

**Published:** 2025-07-10

**Authors:** Alessandra Burgio, Cinzia Castagnaro, Daniele Vignoli, Agnese Vitali

**Affiliations:** Italian National Institute of Statistics, Rome, Italy; Italian National Institute of Statistics, Rome, Italy; Department of Statistics, Computer Science, Applications, University of Florence, Florence, Italy; Department of Sociology and Social Research, University of Trento, Trento, Italy

**Keywords:** age at childbearing, age-specific fertility, Italy, medically assisted reproduction, population-based study, total fertility rate

## Abstract

**STUDY QUESTION:**

What is the contribution of medically assisted reproduction (MAR) to total, age-, and parity-specific fertility in Italy?

**SUMMARY ANSWER:**

MAR contributed 3.7% to Italy’s total fertility rate in 2022 and 5.9% to fertility of first order; MAR’s contribution to fertility reached 16% among women aged 40 +  and 31% among women aged 40 +  at first birth.

**WHAT IS KNOWN ALREADY:**

Demography, particularly via postponement of the age at childbearing for both women and men, plays a role in the diffusion of MAR techniques, and the diffusion of MAR techniques may contribute to postpone the age at childbearing. Recent studies found that the contribution of MAR to fertility rates is remarkable and increases over time in countries such as Czech Republic, Denmark, Australia, and the USA. Italy is a country distinguished by one of the lowest average number of children per woman globally, as well as the highest maternal age at first birth and among the highest shares of births to mothers aged 40 years and over in Europe. No prior study has focused on Italy.

**STUDY DESIGN, SIZE, DURATION:**

This study relies on a unique combination of administrative data sources: the Certificate of Delivery Care Registry dataset based on the entire population of live birth deliveries in Italy in 2022 (N = 393 997), administered by the Ministry of Health; the Register of Live Births to the Resident Population in 2022 (N = 393 333), administered by the Italian National Institute of Statistics; and the resident population by age and sex to identify the female population at risk of having a(n additional) child by age (N = 17 006 665) provided by the Italian National Institute of Statistics. Comparisons are made with the year 2013.

**PARTICIPANTS/MATERIALS, SETTING, METHODS:**

We calculate the age-specific fertility rates (total and by parity) for births conceived via MAR and those conceived naturally. These rates are then utilized to assess the contribution of MAR to total and parity-specific fertility, as well as to the mean maternal age at childbearing. This study is the first estimation of its kind for Italy.

**MAIN RESULTS AND THE ROLE OF CHANCE:**

The contribution of MAR to the total fertility rate (for women aged 15–59 years) in Italy increased from 2.1% in 2013 to 3.7% in 2022. Among women aged 40 + , the contribution of MAR to the total fertility rate increases to 16.2% in 2022, up from 8.6% in 2013. The contribution of MAR to first-order fertility rate increases to 5.9% and it reaches 30.9% among women aged 40–59 years in 2022. The mean age at first childbirth among women who conceived via MAR equals to 37.8, up from 36.0 in 2013, compared to those who conceived naturally at a mean age at first birth of 30.4 in 2013 and of 31.3 in 2022.

**LIMITATIONS, REASONS FOR CAUTION:**

Our approach may underestimate MAR’s contribution to the total fertility rate in Italy: mothers in Italy may be more likely to under-report of MAR-births than in other countries, due to social norms that are more resistant to non-conventional paths to parenthood. Our estimates use unconstrained denominators based on the entire population of women in reproductive age, irrespective of parity, to compute fertility rates because the population of women by age and parity is not available from official statistics. In addition, our estimates are somewhat affected by the possibility that couples who underwent MAR treatment would have eventually conceived spontaneously.

**WIDER IMPLICATIONS OF THE FINDINGS:**

Countries characterized by low and late fertility offer a unique test ground for studying the contribution of MAR to fertility rates. In Italy, a late transition to parenthood among the general population aligns with the late transition to parenthood among mothers who conceived via MAR, mirroring that they seek infertility treatments at a relatively late age. For Italy, it will be important to monitor MAR’s contribution to fertility as a new law came into effect in January 2025, that, by recognizing infertility as a pathology, considerably reduces treatment costs hence likely increases demand for MAR. The extent to which a potentially increased demand will translate into access to treatment is uncertain if additional resources are not made available to expand the health system to meet the expected increased demand.

**STUDY FUNDING/COMPETING INTEREST(S):**

We acknowledge funding from Next Generation EU, in the context of the National Recovery and Resilience Plan, Investment PE8—Project Age-It: ‘Ageing Well in an Ageing Society’ (DM 1557 11.10.2022) and the project ‘ALFA—Aligning Law with Family Arrangements’ funded by Fondazione Cariplo 2021-1321. Open access funding provided by University of Trento within the CRUI-CARE agreement. The views and opinions expressed are only those of the authors and do not necessarily reflect those of the European Union or the European Commission. Neither the European Union nor the European Commission can be held responsible for them. No conflict of interest exists.

**TRIAL REGISTRATION NUMBER:**

N/A.

## Introduction

The trend toward delayed reproduction is among the most significant demographic shifts observed in recent decades. The proportion of births to mothers aged 40 years and over has considerably increased throughout Europe during the second half of the 20th century (Sobotka, 2010; Kocourkova *et al.*, 2014). The postponement of childbearing may affect completed fertility because of the limited time remaining for additional children (Leridon and Slama, 2008) and, because the risk of infertility increases with paternal age (De la Rochebrochard and Thonneau, 2003), may lead to involuntary childlessness (Tanturri and Mencarini, 2008; Tocchioni *et al.*, 2022).

Pregnancy postponement plays a role in the rapid increase in use of various types of medically assisted reproduction (MAR) techniques, which encompasses not only ART treatments such as IVF, ICSI, preimplantation genetic testing, cryopreservation of embryos and gametes but also assisted insemination and hormonal treatments such as ovulation induction or stimulation (Zegers-Hochschild *et al.*, 2017); on the other hand, the diffusion of MAR may contribute to further postponing the age at childbearing (Leridon and Slama, 2008; Sobotka *et al.*, 2008).

Recent studies started to evaluate the contribution of ART to overall current and future fertility in different countries (e.g. Hoorens *et al.*, 2007; Leridon and Slama, 2008; Sobotka *et al.*, 2008; Habbema *et al.*, 2009; Lazzari *et al.*, 2021, 2023; Tierney, 2022; Kocourková *et al.*, 2023). These studies estimated the contribution of ART to the total fertility rates (TFRs, see ‘Method’ section for a definition of TFR) to be in the range of 0.05–0.10 children per woman: 0.058 for Czechia in 2020 (Kocourková *et al.*, 2023), 0.088 for Australia in 2017 (Lazzari *et al.*, 2021), 0.08 after 3 years of full access to IVF in the Netherlands in 2002 (Habbema *et al.*, 2009), 0.023 in the USA in 2020 (Tierney, 2022) and 0.05–0.08 for women born in 1975 in Denmark (Sobotka *et al.*, 2008). Research so far failed to estimate the contribution of MAR, rather than ART, to the TFR, hence possibly underestimating the overall contribution of MAR to fertility. The aim of our study is to measure the contribution of all live births conceived with MAR to the TFR, without excluding births obtained by treatments not classified as ART, to consider all fertility interventions.

No previous study has analyzed the impact of MAR, nor ART, on the TFR in Italy. Italy has the highest maternal mean age at first birth in Europe, reaching 31.8 years in 2023 (Eurostat, 2025). It also has one of the highest incidences of births to mothers aged over 40 years in Europe, both for first births (7.83% compared to a European average of 4.26% in 2022) and for all births (8.99% vs 5.97%) (Eurostat, 2025). Italy makes up over 28% of all births to mothers aged 50 years and over in Europe (492 births in Italy out of 1738 in the EU in 2022) followed by Spain with 15% (Eurostat, 2025). Italy’s TFR decreased from 2.66 children per woman in 1964, the peak year of the Italian post-war baby boom, to 1.19 in 1995. This was the country’s lowest ever recorded value which made Italy one of the world’s forerunners of ‘lowest-low’ fertility, i.e. with an average number of children per woman below 1.3, a level that poses far-reaching demographic, social, and economic consequences for modern welfare states (Kohler *et al.*, 2002). Although an increase was visible during the 2000s, leading to 1.44 children per woman in 2008–2010, the TFR has been decreasing continuously since 2011, reaching a figure of 1.20 children per woman in 2023 and 1.18 in 2024, among the lowest in Europe (ISTAT, 2025). Current fertility levels are hence lower than the historical minimum of 1.19 children per woman in 1995 but the mean age at childbearing (MAC) is now almost 3 years higher, reaching age 32.5 years in 2023 versus 29.8 years in 1995 (ISTAT, 2024). The observed postponement of fertility is accompanied by an increase in childlessness and a decrease in higher-order births, particularly those of third order and above.

The legislation regulating MAR use in Italy dates back to 2004 (law 40/2004). The law was modified several times since then. These legal modifications meant considerable changes in MAR outcomes. For instance, the law originally prohibited the fertilization of more than three oocytes per treatment and imposed the simultaneous transfer of all embryos into the uterus (law 40/2004), leading to a high incidence of multiple MAR-pregnancies and births (Esposito *et al.*, 2024). In 2009, when this aspect of the law was deemed unconstitutional, the share of multiple births on total live births fell considerably (Levi Setti *et al.*, 2011). Current legislation forbids the use of MAR to singles and same-sex couples. Only co-residing married or cohabiting opposite-sex couples can access MAR, provided that both partners are alive and of potentially fertile age. Surrogacy is not available, neither for same-sex nor opposite-sex couples. Given its restrictive access to MAR, Italians resort to cross-border reproductive care in greater numbers compared to other Europeans with less restrictive legislations (Shenfield *et al.*, 2010; Präg and Mills, 2017). At the time of writing, availability of public MAR centers, waiting times, access costs, and age-related criteria for accessing state-subsidized MAR treatments vary across Italian regions. Such variability, which so far has created social inequalities in access to MAR, is bound to be regulated: infertility is officially recognized by the National Health System as a pathology and, as a consequence, from 1 January 2025, MAR was included in the so-called ‘Essential Assistance Levels (LEA)’. This means that homologous fertilization (i.e. when gametes (sperm and eggs) from the couple are used) is included in the services that the National Health System is required to provide to all citizens free of charge. Heterologous fertilization (i.e. when gametes (sperm and/or eggs) from external donor(s) are used) instead can be accessed upon payment of a participation fee of ∼1500 euros (variable across regions). The new legislation applies to heterosexual couples with women aged up to 46 years old.

The international literature has often depicted Italy as ‘the last bastion’ of traditional value orientation (Aassve *et al.*, 2024) due to the dominant role of the Roman Catholic Church and its resistance to modern family changes (e.g. non-marital childbearing and multi-partner fertility) which started to occur in countries with equivalent economic status from the 1960s. Non-marital childbearing is however increasing rapidly and other family-related behaviors are changing (Aassve *et al.*, 2024), including the role of MAR in fertility. Despite a restrictive legislation in Italy and the use of MAR techniques remaining relatively rare in comparison to the potential demand, its utilization has significantly increased over time. Since 2005, when the MAR National Register began its activity, the number of annual treatments almost doubled (from 63 585 in 2005 to 109 755 in 2022), as have MAR-births (from 1.2% in 2005 to 4.2%, equal to about 16 000 births, in 2022), alongside a decrease of the number of women in reproductive age (2.2 million fewer women aged 15–49 years in 15 years). However, the contribution of MAR to the TFR in Italy is unknown. Understanding this is crucial, as the proportion of MAR-births relative to the total number of births reflects the immediate output of reproduction but does not fully reveal underlying health and social challenges. The number of births is determined by both individual reproductive behaviors and the size of the female population of reproductive age—typically defined as women aged 15–49 years old (Preston *et al.*, 2001). Fertility rates, on the other hand, reflect reproductive behavior and choices within a society, as they represent the sum of age-specific fertility rates, that is, the number of births to women of each age relative to the number of women in that age group. Hence, while the number of births merely captures the occurrence of the phenomenon, fertility rates reflect the underlying conditions and capacities shaping reproduction.

This paper assesses, for the first time, the contribution of MAR to fertility rates in Italy, using a unique combination of data sources from the Italian National Institute of Statistics (ISTAT) and the Ministry of Health. More specifically, we examine the contribution of MAR (and ART) to total, age-, and parity-specific fertility rates, as well as to fertility timing.

## Materials and methods

### Data sources and study population

This study focuses on the live births from MAR and non-MAR-related pregnancies, utilizing a strategic combination of administrative data sources. Data on live births by age of mother and birth order stem from the ‘Register of live births to the resident population’ (RLB), provided by the Italian National Institute of Statistics (ISTAT) and collected annually in each Italian municipality since 1999. This register provides demographic statistics used by ISTAT to compute national fertility indicators but it does not collect information on mode of conception.

Individual-level data on MAR-related births in Italy are available via the ‘Certificate of Delivery Care Registry’ (CEDAP) administered by the Ministry of Health, providing information on all births occurring in hospital birth departments in Italy since 1 January 2002. This includes mode of conception and enables differentiation between the following types of MAR treatment: pharmacological treatment for ovulation induction; IUI; Gamete IntraFallopian Transfer; IVF, ICSI and “other techniques”. Information on the mode of conception (whether natural or via MAR) is missing in 0.93% of the total births (such records were excluded from the analysis). Among MAR-births, information regarding the type of MAR treatment used is missing in 1.78% of births. In case of multiple births, information is collected for each birth. The data include MAR-births conceived abroad by Italian residents, provided the births occurred in Italy. The CEDAP certificate is filled by health professionals (midwife, nurse, doctor who attended the birth or doctor in charge of the operational unit in which the birth occurred). Information on the mother’s reproductive history (including parity, live births, stillbirths, previous induced abortions, and previous miscarriages), pregnancy-related information (such as medical examinations, ultrasounds, gestational age, etc.), and use of MAR treatment are provided directly by the woman.

The number of live births to the resident population in Italian municipalities stemming from the RLB data source is slightly lower than the total number of live births occurring in Italy stemming from the CEDAP data source (respectively, 393 333 vs 393 997 in 2022). To combine the two sources, we exclude from the CEDAP source those births to the non-resident population, that hence cannot be present in the RLB source. This exclusion leaves us with 392 823 births to the resident population from the CEDAP source.

To evaluate the contribution of MAR to fertility rates we re-proportioned, i.e. weighted, the number of live births with and without MAR by maternal age and parity obtained from the CEDAP data source according to the total number of live births recorded in the RLB data source. Without re-proportioning the data between the two sources, the TFR calculated using CEDAP data would differ slightly from the TFR derived from RLB data, which is used in official statistics. Therefore, re-proportionating the CEDAP data allows us to accurately determine the contribution of MAR to the TFR reported in official statistics.

Finally, we make use of a third source of administrative data: population estimates of the municipal resident population (i.e. having usual residence in Italy even if temporarily absent, of both Italian and foreign citizenship) by sex and age. From this source, we obtain the estimate of the female resident population in fertile age. For each year *t* and for each age *x* considered in the analysis, the female population at risk is calculated by summing up the population of women in fertile age at 1st of January of year *t* and the corresponding population at 1st of January of year *t + 1*, then dividing by 2. Re-proportioned CEDAP data are hence combined with population estimates of the total female resident population at risk of having a birth by age to compute total, age-, and parity-specific MAR- and non-MAR fertility rates. Our analyses refer to the years 2022, i.e. the latest available from the CEDAP source at the time of writing, and 2013. We chose 2013 as a comparison because in 2014 legal changes took place, e.g. heterologous fertilization became available, which made access to MAR treatments less strict that it previously was.

### Method

The TFR is defined as the average number of children that a woman would bear if she survived through the end of her reproductive life and experienced at each age a particular set of age-specific fertility rates. Following Preston *et al.* (2001), it can be written as:


(1) $$\mathrm{TFR}\;\left[0,T\right]=n\;\;\cdot \;\sum_{x=\alpha }^{\beta -n}{\;}_{n}F_{x}\left[0,T\right]$$

where *x* is the woman’s age, *α* and *β* are the minimum and maximum ages at childbearing, [0, *T]* is the period of time, n is the number of years a woman spends in each *n*-year-wide age interval and ${}_{n}F_{x}$ is the annual rate at which a woman bears children, and can be expressed as follows:


(2) $${}_{n}F_{x}\left[0,T\right]={}_{n}B_{x}{/}_{n}W_{x\cdot }$$

In other words, ${}_{n}F_{x}$ is the age-specific fertility rate relating the number of births occurring to mothers aged *x* to *x* + *n* (${}_{n}B_{x}$) in the period 0 to *T* with the number of person-years lived by women in that age interval, approximated by the mid-year population of women, i.e. the average between the number of women in each age interval who are alive at the beginning and at the end of the time period (${}_{n}W_{x}$).

Because we rely on annual data, [0, *T]* refers to periods of 1 year and *n* = 1. The TFR is usually computed considering 15 and 50 as the minimum and maximum ages at childbearing, respectively, as the vast majority of births occur to women in this age range (Preston *et al.*, 2001). Hence, we can simplify the formulas for the TFR for a given year and the age-specific fertility rate, respectively, as follows:


(3) $$\;\mathrm{TFR}=\overset{49}{\underset{x=15}{\;\sum }}\;F_{x}$$

(4) $$F_{x}=B_{x}/W_{x\cdot }$$

We then construct the TFR based on the method of conception distinguishing between MAR and non-MAR. First, we compute age-specific fertility rates for MAR- and non-MAR-related conceptions as follows:


(5) $${}^{\mathrm{MAR}}F_{x}={}^{\mathrm{MAR}}B_{x}/W_{x}$$

(6) $${}^{\mathrm{nonMAR}}F_{x}={}^{\mathrm{nonMAR}}B_{x/}W_{x}$$

where ${}^{\mathrm{MAR}}B_{x}$ and ${}^{\mathrm{nonMAR}}B_{x}$ are the number of live births conceived, respectively, after receiving MAR treatment and naturally, by mothers aged *x* in a given year, while $W_{x}$ is the number of resident women aged *x* in the year. Following the standard procedure adopted by official statistics in Italy, $W_{x}$ is estimated by averaging the population of women at age *x* at the end of the year and the respective population at the end of the following year (i.e. the mid-year population).

The TFR (7) and its decomposition into its MAR- (8) and non-MAR (9) variants are computed as follows:


(7) $$\mathrm{TFR}={\;}^{\mathrm{MAR}}TFR+{\;}^{\mathrm{nonMAR}}TFR$$

(8) $${}^{\mathrm{MAR}}TFR=\sum_{x=15}^{49}.^{\mathrm{MAR}}F_{x}$$

(9) $${}^{\mathrm{nonMAR}}TFR=\sum_{x=15}^{49}.^{\mathrm{nonMAR}}F_{x}$$

MAR and non-MAR age-specific fertility rates by order k, with *k * =  1 for first child, 2 for second child and 3 for third or higher-order child, are computed as follows:


(10) $$F_{x}{}^{k}={}^{\mathrm{MAR}}F_{x}{}^{k}+{}^{\mathrm{nonMAR}}F_{x}{}^{k}$$

(11) $${}^{\mathrm{MAR}}F_{x}{}^{k}={}^{\mathrm{MAR}}B_{x}{}^{k}/W_{x}$$

(12) $${}^{\mathrm{nonMAR}}F_{x}{}^{k}={}^{\mathrm{nonMAR}}B_{x}{}^{k}/W_{x}$$

Official statistics for Italy do not provide information on the population of women by age and by parity. This means that our denominators in the fertility rates are unconstrained (i.e. they refer to the entire population of women by age, irrespective of parity).

Accordingly, MAR and non-MAR TFRs by order k are computed as follows:


(13) $$TFR^{k}={}^{\mathrm{MAR}}TFR^{k}+{}^{\mathrm{nonMAR}}TFR^{k}$$

(14) $${}^{\mathrm{MAR}}TFR^{k}=\;\sum_{x=15}^{49}{\;}^{\mathrm{MAR}}F_{x}{}^{k}$$

(15) $${}^{\mathrm{nonMAR}}TFR^{k}=\;\sum_{x=15}^{49}{\;}^{\mathrm{nonMAR}}F_{x}{}^{k}$$

Since the aim of our study is to measure the contribution of MAR to the TFR, it is particularly important to include maternal ages which are higher than the conventional upper limit used in demographic research, i.e. age 50 years. In our population, age 60 years captures the actual upper age of mothers in the official birth register in 2022. Indeed, out of the 492 births to women aged 50+ observed in 2022, 363 were conceived via MAR (74%). Hence, we also compute the MAR and non-MAR TFR using the age range 15–59 years for women. In this way, we ensure comparability with previous studies and with the usual age range (15–49 years) used to compute the TFR (including official statistics for Italy), but interested readers can also gather information on the contribution of MAR to the TFR when extending the age range to consider older ages for which a birth is observed in official statistics (15–59 years).

Fertility timing is measured through the MAC, distinguishing between its MAR and non-MAR components, as follows:


(16) $$\mathrm{MAC}=\overset{49}{\underset{x=15}{\;\sum }}x\times F_{x}/\sum_{x=15}^{49}F_{x}$$

(17) $${\;}^{\mathrm{MAR}}MAC=\sum_{x=15}^{49}x\times {}^{\text{MAR}}F_{x}/\sum_{x=15}^{49}{}^{\text{MAR}}F_{x}$$

(18) $${}^{\mathrm{nonMAR}}MAC=\sum_{x=15}^{49}\;x\times {}^{\mathrm{nonMAR}}F_{x}/\sum_{x=15}^{49}{}^{\mathrm{nonMAR}}F_{x}$$

Formulas 16, 17, and 18 are also applied to the first birth order, using the first-order age-specific fertility rates ($F_{x}{}^{1}$), and to the age range 15–59 years.

Finally, for comparability with previous studies, we also provide estimates for the sub-group of births conceived via IVF and ICSI, amounting to 47.8% and 35.0%, respectively, of the total MAR-births. In this way, we provide a separate estimate of the contribution of ART to the TFR.

## Results

### The contribution of MAR to fertility quantum and timing in Italy

The contribution of MAR to the intensity (total and of first order) of childbearing is reported in Table 1. Among women aged 15–49 years old, the contribution of MAR to Italy’s TFR equals to 3.6% in 2022, i.e. 0.045 children out of a total of 1.242 children per woman, almost double the value of 2.1% in 2013, i.e. 0.029 out of 1.388 children per woman. The contribution of MAR to the TFR jumps to 15.5% for women aged 40–49 years, a substantial increase from the 8.2% observed in 2013.

**Table 1.** Contribution of MAR to Italy’s TFR and mean age at childbirth, by total and first-order fertility for women aged 15–49 years and women aged 15–59 years old, 2013 and 2022.

	Age 15–49 years				Age 15–59 years			
	Total		First order		Total		First order	
	2013	2022	2013	2022	2013	2022	2013	2022
Fertility rate	1.388	1.242	0.695	0.614	1.389	1.243	0.696	0.614
of which non-MAR fertility rate	1.359	1.197	0.672	0.578	1.359	1.197	0.672	0.578
of which MAR fertility rate	0.029	0.045	0.023	0.036	0.029	0.045	0.023	0.036
Contribution of MAR to TFR (%)	2.1	3.6	3.3	5.8	2.1	3.7	3.4	5.9
women aged 40+ (%)	8.2	15.5	16.3	30.0	8.6	16.2	16.7	30.9
Mean age at childbirth	31.4	32.4	30.6	31.6	31.5	32.4	30.6	31.7
Mean age at non-MAR childbirth	31.3	32.2	30.4	31.3	31.3	32.2	30.4	31.3
Mean age at MAR childbirth	36.1	37.6	35.9	37.5	36.2	37.8	36.0	37.8

Note: We report fertility rates approximating numbers to three digits. Due to this approximation, the total and first-order fertility rates (reported in the first raw) in some cases do not perfectly equate the sum of non-MAR and MAR fertility rates. MAR, medically assisted reproduction; TFR, total fertility rate; MAC, mean age at childbirth (in years).

The MAC among women aged 15–49 years giving birth to a child (of any parity) after receiving MAR treatment, was 37.6 years while this was 32.2 years among those who conceived naturally. In 2013, the MAC equaled to 36.1 and 31.3 years, respectively. The MAC for MAR-births is 5.4 years higher than the MAC for non-MAR births in 2022 but just 4.8 years in 2013.

**Figure 1.**
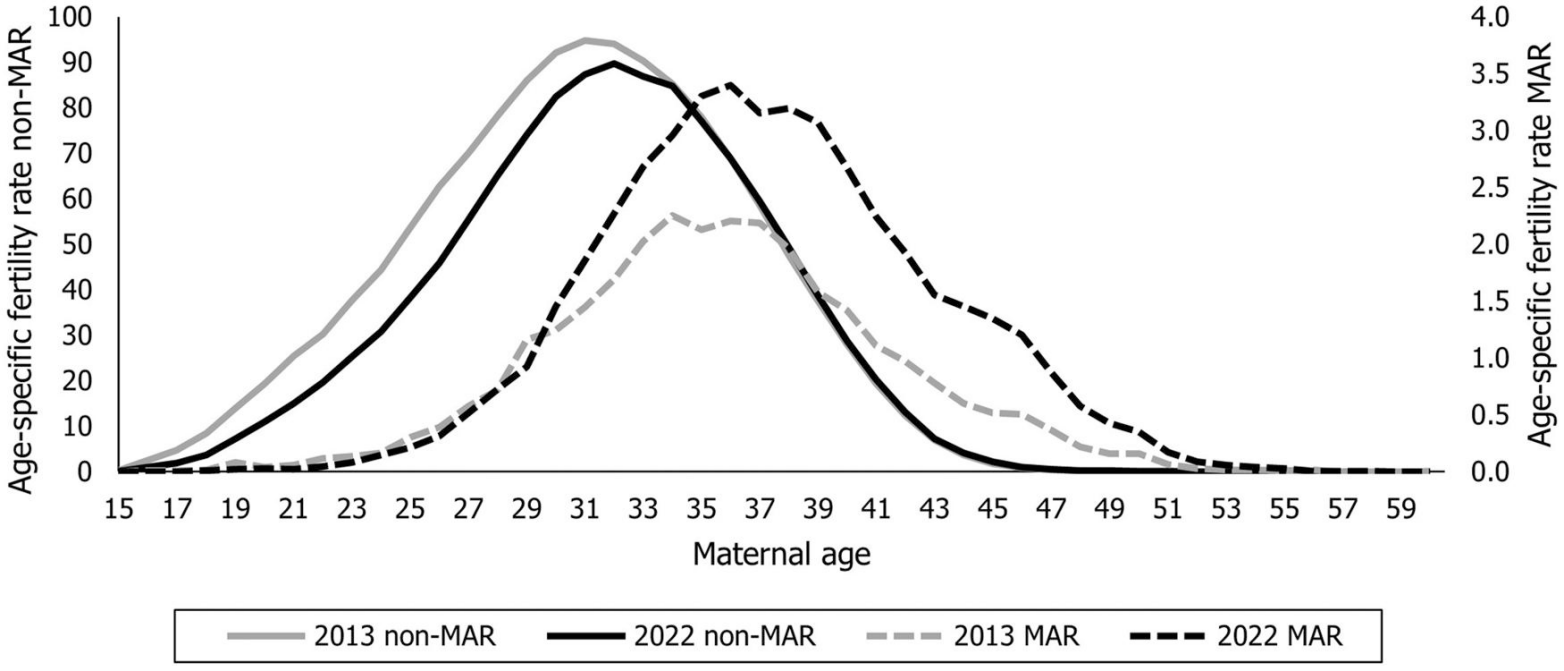
Age-specific medically assisted reproduction (MAR) and non-MAR fertility rates, 2013 and 2022.

The substantial age gap between mothers who conceived naturally compared to those undergoing MAR is the result of the strongly postponed reproductive calendar of mothers, shown in Fig. 1. A shift toward higher maternal ages occurred between 2013 and 2022 in both the MAR and non-MAR groups of mothers, but it is especially pronounced for MAR-fertility between age 40 and 50 years.

Such postponement suggests to also consider, at least in the case of Italy and other countries with late mean age at first birth, a higher upper age limit for women of childbearing age. If we increase the age range to age 59 years, the contribution of MAR to the TFR increases to 3.7% i.e. 0.045 out of 1.243 children per woman and it reaches 16.2% for women aged 40 years and over in 2022, up from 8.6% in 2013 (Table 1). Unsurprisingly, when we consider women aged 15–59 years old, the MAC for MAR-births increases to 37.8 years in 2022 (compared to 37.6 for women aged 15–49 years), and the gap between the MAC for MAR-births and the MAC for non-MAR births increases to 5.6 years; the latter age gap was 4.9 years in 2013 (Table 1).

**Figure 2.**
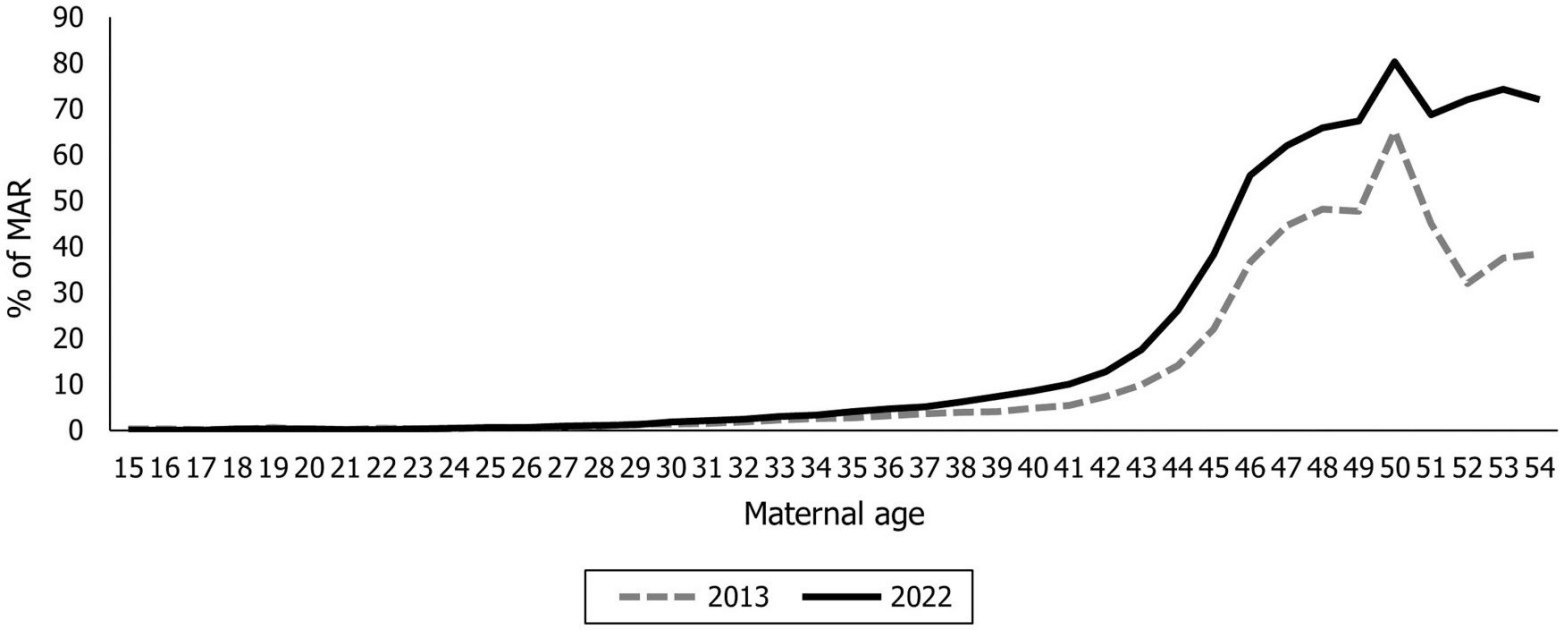
Contribution of medically assisted reproduction (MAR) to age-specific fertility rates (%), 2013 and 2022.

The contribution of MAR to the TFR increases steeply after age 40 years (Fig. 2). The share of the TFR attributable to MAR after age 40 years, 16.2% in 2022, is higher in Italy than previously measured for other settings characterized by an earlier reproductive calendar, e.g. 12% in Czechia in 2020 (Kocourková *et al.*, 2023).

### The contribution of MAR to TFR by birth order

The overall contribution of MAR to the fertility rate of first-order (i.e. related to first births) equals to 5.8% among women aged 15–49 years in 2022, up from 3.3% in 2013 and 5.9% among women aged 15–59 years, up from 3.4% in 2013 (Table 1). The contribution of MAR to the TFR is 30% among first-time mothers aged 40–49 years and 30.9% among those aged 40–59 years in 2022, which is almost double than in 2013 (Table 1).

A total of 80% of MAR-births are first births, equivalent to more than 13 000 live births. Relatedly, the average age of first-time mothers in the case of MAR-births equals to 37.5 years (37.8 if we consider women aged 15–59 years), a value that (almost) coincides with the MAC of MAR-mothers (of any parity): MAR almost entirely concerns first births. The mean age at first birth among mothers who conceived naturally instead equals to 31.3. There is a 6.2-year gap between the mean age at first birth for women who conceived naturally and those who conceived via MAR. Such a gap in age further increases to 6.5 years when we extend the maternal age range to 15–59 years. These numbers confirm previous findings (e.g. Lazzari *et al.*, 2021): MAR-births contribute to raising the overall mean age at first birth. In particular, MAR-births contribute to increase the mean maternal age at first birth by 0.4 years (from 31.3 years among first-time mothers who conceived naturally between ages 15 and 59 years to 31.7 years among all mothers).

**Figure 3.**
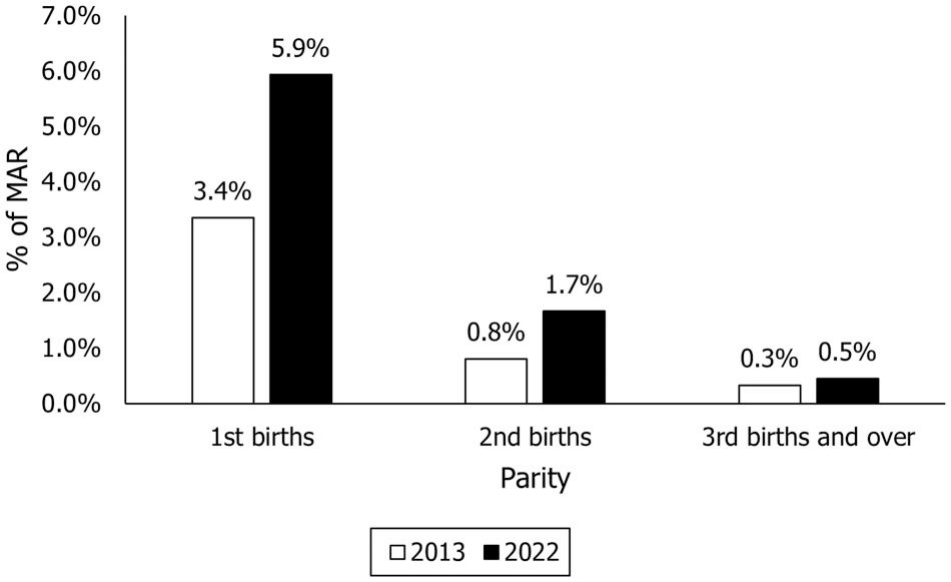
Contribution of medically assisted reproduction (MAR) to fertility rates by parity (first, second, and third or higher-order birth, %), 2013 and 2022.

Figure 3 shows that the contribution of MAR to the fertility rate of second-order (i.e. related to second births) is only 1.7% among women aged 15–59 years in 2022 (1.6% among women aged 15–49 years), though this represents an increase from 0.8% in 2013. The contribution of MAR to the fertility rate of third-order and above is as little as 0.5% (0.4% for women aged 15–49 years), similar to the 2013 figure of 0.3%.

### The contribution of ART to TFR

For comparability with previous studies which have estimated the contribution of ART to the TFR for other countries, Table 2 reports the contribution of the fertility of women aged 15–49 years who received treatment for IVF or ICSI to the total and first-order fertility rate in Italy. ART contributes 0.036 children to Italy’s TFR, i.e. 2.9% of total fertility in 2022, up from 0.021, i.e. 1.5% in 2013. The contribution of ART to the TFR of women aged 40–49 years increases to 12.3%. ART’s contribution to the first-order fertility rate of women aged 40–49 years reaches 25.3%. The mean age at birth (of any parity) and at first birth for mothers who were treated for IVF or ICSI equal to 37.7 and 37.6, respectively, in 2022, in both cases 0.1 years higher compared to when we also include other non-ART techniques.

**Table 2.** Contribution of assisted reproduction technologies to Italy’s TFR and mean age at childbirth, by total and first-order fertility for women aged 15–49 years, 2013 and 2022.

	Total		First order	
	2013	2022	2013	2022
Fertility rate	1.388	1.242	0.695	0.614
of which ART fertility rate	0.021	0.036	0.017	0.029
Contribution of ART to TFR (%)	1.5	2.9	2.5	4.8
women aged 40+	6.2	12.3	13.3	25.3
Mean age at childbirth	31.4	32.4	30.6	31.6
Mean age at ART childbirth	36.6	37.7	36.4	37.6

Note: We report fertility rates approximating numbers to three digits. Due to this approximation, the total and first-order fertility rates (first raw) in some cases do not perfectly equate the sum of non-MAR and MAR fertility rates. ART, assisted reproduction technologies, i.e. IVF and ICSI; TFR, total fertility rate; MAR, medically assisted reproduction.

## Discussion

Our results show that the contribution of MAR to the TFR is substantial in Italy, accounting for 3.6% when using the conventional maternal age range of 15–49 years and 3.7% when extending the age range to 59 years, to include births occurring to older mothers. MAR’s contribution to the TFR increased remarkably between 2013 and 2022, although it remains lower than in other countries. Without the contribution of MAR, Italy’s TFR would have dropped from 1.24 to 1.20 in 2022. The contribution of MAR to the fertility rate of first-order births is 5.8% for the age range 15–49 years and 5.9% for the age range 15–59 years. Remarkably, nearly one in three first-order live births to mothers aged 40 years and over is attributable to MAR. Because 80% of MAR-births are first births, the availability of MAR considerably contributes to reduce involuntary childlessness in Italy.

While the overall results change relatively little when comparing the age ranges 15–49 and 15–59 years, the difference is nonetheless present and pronounced for the sub-population of women who had their first birth at age 40 years or older. For this group, the difference amounts to 0.01 in terms of TFR and 0.09 in terms of age at birth. Future estimates of MAR and ART contributions to the TFR in other countries should systematically consider age ranges extending beyond age 49 years.

The paradox of Italy being caricatured as a bastion of traditionalism in relation to family formation (cf Aassve *et al.*, 2024), while simultaneously experiencing low fertility rates and a rising age at first birth, makes the absence of Italy into the international debate on assisted reproduction particularly striking. Our study addresses this oversight. This is the first study that sheds light on the contribution of MAR (and ART) to total, age-, and parity-specific fertility rates in Italy, highlighting the importance of MAR for the recovery of births at older reproductive ages and enabling the transition to parenthood among infertile couples in a context characterized by one of the lowest fertility levels globally, as well as the highest maternal mean age at first birth and among the highest shares of births to mothers aged 40 years and over in Europe.

Our evidence corroborates previous findings based on different countries showing that fertility over 40 is dominated by MAR (see, e.g. Tierney and Cai, 2019; Lazzari *et al.*, 2023). International research documents that women and men may be unaware of biological fertility age limits and that, if women knew that their fertility was diminishing, they may alter their choices (Seifer *et al.*, 2015). Our results for Italy offer some points for reflection. Success rates of MAR treatments considerably decline with both maternal (Vitagliano *et al.*, 2023) and paternal (Murugesu *et al.*, 2022) age, indicating that, for each MAR-birth, there are couples who did not succeed in becoming parents or having another child. Furthermore, the share of women aged over 40 years who begin MAR treatment with their own gametes is among the highest in Europe (equal to 34.4% in 2021 according to Ministry of Health, 2023 vs a European average of 21% as of 2018 according to Wyns *et al.*, 2022), while the waiting time between first medical consultation for infertility and first consultation in an infertility center increases with the man’s age (Costa *et al.*, 2013), further reducing the chances of MAR-birth at later ages. By promoting early intervention and comprehensive fertility management, it may be possible to improve treatment outcomes and mitigate the pressure on MAR services. Education programs to increase awareness regarding reproductive health and infertility prevention are needed as well as strengthening health systems to enable informed reproductive decisions (Ziebe and Devroey, 2008; Mburu *et al.*, 2023) and more so in Italy, a country with the highest gap between desired and actual fertility in Europe (Beaujouan and Berghammer, 2019). There is a pressing need to foster a broader culture of fertility awareness, for both men and women (Ferlin *et al.*, 2022).

On the one hand, if access to MAR was expanded in the future, MAR’s contribution to the TFR may increase. This consideration is timely given that, with the introduction of the ‘Essential Assistance Levels’ for MAR on 1 January 2025, heterosexual couples will have the right to access treatments up to age 46 years for women for free if accessing homologous fertilization or with the payment of a standard amount if accessing heterologous fertilization with egg and/or sperm donation. The new law considerably reduces and standardizes the cost of accessing MAR for heterosexual couples throughout the country. As a consequence, a notable increase in the number of couples seeking MAR treatments is likely in the coming years, potentially involving couples seeking treatment to conceive a second or higher-order child. On the other hand, it is worth to note that a future expansion of MAR may lead couples to further delay childbearing, therefore possibly negatively impacting the TFR.

In any event, there are concerns that the current infrastructure may not be adequate to meet the growing demand for MAR techniques, particularly in regions where facilities are sparse. Public centers, which have so far handled 42% of the MAR cycles, are unlikely to be able to absorb the entire demand, highlighting the need for expanded capacity or alternative solutions.

Although our analysis is based on the best available data for Italy with comprehensive territorial coverage, our estimates are somewhat affected by the possibility that some couples who underwent MAR treatments would have eventually conceived spontaneously, leading to a potential overestimation of MAR’s true impact on fertility levels. We share this limitation with earlier studies for other countries (see, e.g. Lazzari *et al.*, 2023). However, our estimates may also underestimate MAR’s contribution. There may be underreporting of MAR-births among mothers in Italy, possibly due to social norms that are more resistant to non-conventional paths to parenthood compared to other countries. This is reflected in the relatively slow adaptation of the legislation supporting assisted reproduction (e.g. heterologous fertilization was only legalized in 2014). Finally, our estimates use unconstrained denominators to compute fertility rates, i.e. the entire population of women in reproductive age, irrespective of parity, because the population of women by age and parity, which is required to compute constrained rates, is not available from official statistics. Future attempts to estimate MAR’s and ART’s contribution to fertility in other countries with available data should aim to use constrained fertility rates and consider age ranges extending beyond age 49 years.

Previous research has largely focused on ART alone, failing to capture the full scope of MAR’s contribution to fertility rates, thereby potentially underestimating its demographic impact. Our study addresses this gap by offering a comprehensive assessment of all fertility interventions to fertility in Italy. With recent estimates indicating that one in six people will experience infertility during their lifetime (WHO, 2023), and given the growing reliance on MAR alongside the trend of delayed childbearing, MAR is set to play an increasingly pivotal role in shaping future fertility patterns (see, e.g. Tierney, 2022; Lazzari *et al.*, 2023). This is particularly true for advanced maternal ages in low-fertility countries, extending well beyond Italy to other wealthy nations.

## Data Availability

The data underlying this article cannot be shared publicly due to sensitive data that can be accessed under formal authorization of data owner. The data will be shared on reasonable request to the corresponding author.
